# Event and Comment

**Published:** 1909-08-15

**Authors:** 


### DENTAL REGISTER.

**Vol. LXIII. August 15, 1909. No. 8.**

### EVENT AND COMMENT.

INCREASING OUR LITERARY OUTPUT. The President of the Illinois State Dental Society, in his annual address this year, recounting the work done by the Executive officers during the past year, referred especially to the results of organizing a course of home study, intended to do the work of a scholastic post graduate course. He predicted as a result of this endeavor that it was possible that three thousand original papers on dental topics would be written by dentists of Illinois this year. This statement seems somewhat exaggerated, in view of the fact that there are only 3,000 dentists in Illinois, and only about half of them are members of the State Society. It may be practicable to get an average of two papers from each of the 1,500 members of the Illinois State Society, but we very much doubt it, and it is also questionable whether papers written upon urgent request and by men not accustomed to do such things would be of sufficient value to interest dental society members. These papers would be either very elemental and lacking in systematic instruction or would have no great value in advancing professional literature, at the same time it is a stupendous achievement even should the President's predictions be liberally discounted. If the profession of Illinois should produce 300 papers on 300 dental subjects during any year it would mean an estimate of 15,000 pages of printed matter, and the Dental Review which prints the transactions of these societies would have to be enlarged one half or print semi-monthly. Its a grand thing to get the profession to working, but it would be much better could we adopt some plan of stimulating research and culture.

